# De-implementation of detrimental feeding practices: a pilot protocol

**DOI:** 10.1186/s40814-020-00720-z

**Published:** 2020-11-19

**Authors:** Taren Swindle, Julie M. Rutledge, Susan L. Johnson, James P. Selig, Geoff M. Curran

**Affiliations:** 1 Department of Family and Preventive Medicine, University of Arkansas for Medical Sciences, 4301 W. Markham St, #530, Little Rock, AR 72205-7199 USA; 2 College of Applied and Natural Sciences, Louisiana Tech University, Ruston, USA; 3 Department of Pediatrics, University of Colorado School of Medicine, Aurora, USA; 4 College of Public Health, Department of Biostatistics, University of Arkansas for Medical Sciences, Little Rock, USA; 5 Department of Pharmacy Practice and Psychiatry, University of Arkansas for Medical Sciences, Central Arkansas Veterans Healthcare System, Little Rock, USA

**Keywords:** De-implementation, Childcare, Feeding practices, Nutrition, Implementation science

## Abstract

**Background:**

Early childhood educators (ECEs) often use detrimental feeding practices and are slow to implement positive feeding practices. Nevertheless, few studies have aimed to understand and change ECEs’ feeding practices. This gap needs to be addressed because implementation (i.e., adding new, evidence-based practices) and de-implementation (i.e., stopping low-value or harmful practices) are distinct processes that require unique strategies.

**Methods:**

We will develop a de-implementation strategy for detrimental feeding practices using evidence-based quality improvement (EBQI) sessions to engage stakeholders and draw on the Niven process model for de-implementation. Then, we will investigate the effects of the de-implementation strategy in a proof-of-principle study. The de-implementation strategy will be evaluated in 2 partnering childcare agencies using a pre-post, within-site design. For our primary outcome, we will interview educators throughout the school year to assess the feasibility and acceptability of the intervention and survey them with standard measures for assessing feasibility and acceptability. For secondary outcomes, we will investigate its effects on the use of detrimental and evidence-based feeding practices by teachers and impacts on child BMI and diet.

**Discussion:**

The current study will establish the feasibility and acceptability of our de-implementation approach and will provide preliminary data toward 3 predicted secondary outcomes: (1) decreased detrimental feeding practices by ECEs, (2) increased adoption of and fidelity to nutrition promotion practices, and (3) improved child dietary outcomes. These results are expected to contribute to the uptake and sustainability of mealtime interventions to improve the diets of young children. Results will also apply to the field of implementation science by informing processes for developing de-implementation approaches in a community setting.

## Background

Diet quality predicts a child’s physical, mental, and academic well-being [1–3], yet the diets of many children in the USA are of low nutrient density [4, 5]. One important setting for addressing children’s dietary quality is childcare given that 11 million children under age 5 are in childcare settings in the USA [6] and may consume over two-thirds of their daily diets in this setting [7]. Research supports that childcare influences children’s diets more broadly with spillover effects into the home [8, 9]. Further, dietary habits established in childhood persist into adulthood [10–12]. Thus, promoting healthy habits at childcare has both immediate and lifelong consequences.

Early childhood educators (ECEs) are influential adults in the childcare setting. Evidence-based feeding practices performed by ECEs that positively influence a child’s diet include role modeling [13–16], cuing children to hunger and satiety [13, 14, 16–18], discussing and offering new foods [19–21], and facilitating the exploration of foods [22, 23]. These practices are associated with children’s increased self-regulation, fewer rejections of healthy foods, and increased willingness to try new foods [14, 24–27]. These findings illustrate the positive impact adults can have on children when evidence-based feeding practices are implemented.

Detrimental feeding practices have also been identified [14, 15, 24, 28], and unfortunately, ECEs often use detrimental feeding practices and are slow to implement positive ones [19, 29, 30]. Detrimental feeding practices observed in childcare include hurrying children through the meal [13, 31] and coercing them to eat more [13, 31–36]. Observational research on childcare meals reveals that ECEs frequently pressure children to eat more food regardless of hunger state (19%), hurry children to finish or “be done” (32%), and coerce children to eat certain foods (30%) [18]. Detrimental practices are associated with overeating, long-term food rejections, and decreased intake of healthy foods [24, 29, 37]. Additional effects of detrimental feeding practices include higher child weight-for-height percentiles [38], diminished self-regulation [14, 25, 39], and decreased preference for and intake of fruits and vegetables [21, 24, 32, 39]. The frequency of such detrimental feeding practices and their role in driving unhealthy eating behaviors indicate a need for intervention.

Reports by ECEs reveal that there is wide variation in the frequency and content of training about nutrition and feeding practices, how the training is delivered, and the qualifications of instructors [40–42]. Previous interventions that targeted ECEs include tailored training and provision of resources (e.g., policies and curricula) [43], short didactic courses [44], and web-based self-assessment tools with tailored recommendations and supports [45]. No studies have documented a significant reduction in the observed use of detrimental feeding practices by ECEs. Further, although accredited bodies have established guidelines for structuring meals (e.g., family-style dining and child-sized utensils) [13] and informing the goals of meals (e.g., promoting children’s choice) [46], there are no similar guidelines to address the prevalent, detrimental feeding practices of ECEs. Therefore, a critical barrier to success in this area is the lack of effective strategies to stop detrimental practices.

*Implementation Science* (IS) provides a promising approach to address the need to reduce, remove, and/replace, detrimental feeding practices in children. Inherent to IS is the concept of stopping practices that are not evidence-based, particularly those that are harmful or costly—this is the goal of a de-implementation [47]. Such an approach has been applied to medical service overuse with the aims of reducing low-value services and overtreatment and increasing the use of evidence-based guidelines [48, 49]. De-implementing detrimental practices is distinct from implementing evidence-based practices [50]; the process can be more difficult and require more intense strategies [50]. De-implementation “is far more than merely reversing the implementation process” [51]. While medical communities have developed low-value lists (e.g., Choosing Wisely [51–53]) to prioritize patient-care practices that are ineffective or harmful [48, 54], simply disseminating recommendations for de-implementation has limited impact [55]. An added intervention is usually needed [56]. In clinical practice, the most effective strategies for de-implementation are new policies and changes to funding structures [56]. With ECEs’ feeding, however, these types of top-down, control-based extrinsic motivators are not a realistic approach because ECEs feeding practices are not highly monitored and because these settings are often under-resourced. Rather, effective strategies to shift intrinsic motivation are likely needed. Thus, research is necessary to formulate effective de-implementation strategies in a community setting.

Previous studies by the study team highlight both individual-level and contextual factors that impact ECE feeding practices. For example, detrimental practices are more common in a cafeteria setting with crowded, inflexible schedules and in the presence of competing foods from home [57]. Additionally, teachers who use detrimental feeding practices frequently are also significantly less likely to adhere to other nutrition promotion practices (i.e., hands-on exposures). Interviews conducted with ECEs explored how cultural factors (both personal and professional) impact feeding practices with children and identified over 20 discrete barriers and facilitators, including a prevalent concern about child food insecurity that educators felt responsible to address, an emphasis on manners, and varying beliefs about encouraging children to try foods [58]. These data provide a solid understanding of factors influencing feeding practices from which to work to remove detrimental practices.

### Study aims

In an ongoing trial, we are applying principles of *Implementation Science* (IS) to increase ECE adoption of evidence-based nutrition promotion practices through the WISE (Together, We Inspire Smart Eating) intervention (described below in detail). However, the ongoing study does not explicitly attempt to decrease routinized, detrimental feeding practices. This gap needs to be addressed because implementation (adding new, evidence-based practices) and de-implementation (stopping low-value or harmful practices) are distinct processes that require unique strategies [50, 51]. We propose that each may independently predict eating behaviors. The planned study will explore an innovative de-implementation approach aimed to reduce detrimental feeding practices by ECEs. This is distinct from ongoing work aimed at increasing implementation of WISE evidence-based practices without attention to de-implementation [59].

*Specific aim 1*. Develop a de-implementation approach for detrimental feeding practices. We will use evidence-based quality improvement (EBQI) sessions [60–62] to engage stakeholders (parents, educators, and administrators) to develop a socio-culturally informed de-implementation approach for detrimental feeding practices. Qualitative data on barriers to evidence-based feeding practices and facilitators of detrimental ones (collected prior) will inform stakeholder-selected strategies.

*Specific aim 2*. Investigate the feasibility, acceptability, and preliminary effects of the de-implementation approach in a proof-of-principle study. The de-implementation approach will be evaluated in 2 partnering childcare agencies using a pre-post, within-site design [63]. The primary outcome will be feasibility and acceptability, which we will assess through qualitative interviews and educator surveys. Second outcomes will include measures of preliminary effectiveness including effects on the use of detrimental and evidence-based feeding practices by teachers and impacts on child body mass index (BMI) and diet. We will combine these data with data from an ongoing trial [59] to compare (a) basic WISE implementation, (b) enhanced WISE implementation, and (c) enhanced WISE implementation with de-implementation support that targets detrimental feeding. This is the focal aim of our study.

## Methods

### Aim

This project will enhance the overall objective of an ongoing trial [59] to develop and test implementation strategies that are designed to increase the adoption of 4 evidence-based components of the WISE intervention, which aims to prevent obesity and promote nutrition among children. Our data suggest that *positive feeding practices* is the only component of WISE that competes against detrimental, routinized practices. Thus, this study will pursue a de-implementation approach to decrease detrimental feeding practices.

### Setting and participants

Lincoln Parish Head Start (15 classrooms) is primarily African American (ECEs, 97%; families served, 87%). Lincoln Parish Early Childhood Center (LPECC, 12 classrooms) serves families who are white (52%), African American (40%), or Hispanic (5%). ECEs at LPECC are white (58%) and African American (42%).

### Intervention

The WISE components [64] include (1) multiple hands-on exposures to fruits and vegetables, (2) use of a mascot puppet to promote fruits and vegetables to children, (3) appropriate role modeling by ECEs, and (4) positive ECE feeding practices. WISE lessons occur during classroom instruction time, and the training encourages ECEs to use WISE components 2 through 4 at meals as appropriate. The focus of the current study is on developing and testing strategies to decrease detrimental feeding practices which are hypothesized to compete with positive feeding practices.

### Design

Our proposed method to develop and test a de-implementation approach to reduce detrimental feeding practices in the childcare setting will draw on (1) the Niven model of de-implementation [56], that proposes an iterative method by which stakeholders and practitioners partner to tailor, evaluate, and sustain a de-implementation approach; (2) salient theoretical domains in behavior change theories for designing implementation strategies [65] that draw on theories of *motivation* (e.g., social learning theory), *action* (e.g., cognitive behavioral theory), and *organization* (e.g., diffusion theory) and that align well with the categories of possible implementation strategies outlined by Powell et al. [66]; and (3) the RE-AIM evaluative framework [67], that provides a framework to assess outcomes of implementation and to guide measurements [68]. The integration of these approaches will provide a sound foundation for the proposed research.

#### Aim 1

Engaging stakeholders in the selection and tailoring of de-implementation strategies to comprise a comprehensive approach is central to the Niven process model of de-implementation [56]. We are operationalizing this process with an EBQI panel approach [60–62], which will operate according to principles of community-based participatory research [69] and best practices for engaging stakeholders in IS [70]. Directors, ECEs, and parents will contribute local knowledge needed to tailor strategies to their own contexts, while implementation experts (i.e., the research team) will contribute knowledge on materials, procedures, and tools needed for successful implementation. All members share decision-making power about prioritizing barriers and selecting implementation strategies to target those barriers. EBQI panels have been used to produce effective implementation strategies [71–73], while also promoting buy-in from implementing organizations and fostering beneficial partnerships between implementers and researchers [74].

The EBQI process will (1) prioritize barriers and facilitators to stopping detrimental feeding, (2) identify theoretically informed de-implementation strategies and match to priorities, and (3) tailor de-implementation strategies to the early childhood context. The panel will consider strategies that will promote sustainability to prevent reversion to detrimental feeding practices [56]. The panel will include at least 8 stakeholders from the partnering agencies and 2 stakeholders from an existing EBQI panel who will act as mentors to other stakeholders as needed. The diversity of the EBQI will reflect the diversity of the local context.

EBQI is a flexible process conducted across a series of meetings with topic-driven agendas; each session will last 2 hours. *In EBQI session 1*, the research team will present a summary of findings related to school-based feeding practices from the ongoing trial, conduct a “member checking” exercise with participants to check the validity of these findings, and reach consensus on key barriers and facilitators that will drive selection of the de-implementation strategies. *In EBQI session 2*, we will present potential implementation strategies mapped by the research team to the Expert Recommendations for Implementing Change (ERIC) [75] and taxonomy of implementation strategies with consideration of the theoretical domains of behavior change [65]. To reach a consensus on the implementation strategies, we will use techniques outlined by Powell et al. [76], including concept mapping. This method provides quantifiable information and promotes efficient collection of input in real time. *In EBQI session 3*, we will present the draft strategies/tools, collect feedback for revisions, and receive final approval to pilot test them. The research team will take 1 month to develop and finalize the strategies/tools selected by the group. *In EBQI sessions 4*, we will launch a pre-test of the materials in a small number of classrooms in which the stakeholders teach. *In EBQI session 5*, we will gather feedback from stakeholders about the feasibility and acceptability of the de-implementation strategy to inform iterations and improvements to the approach.

#### Aim 2

After developing the de-implementation approach with stakeholders (aim 1), the next step in the Niven model of de-implementation [56] is to evaluate the process and impact of the approach. During summer professional development days, a face-to-face five hour training will be held with the ECEs, administrators, and key staff of the centers. The training will be led by the lead study investigators. The training will focus on teaching the attendees about the new approach that is developed from the EBQI sessions and practicing with the materials.

Using the RE-AIM evaluation framework (see Table 1), we will collect data focused on de-implementation outcomes as our primary outcome. Specifically, we will collect surveys, which will include the Acceptability of Intervention Measure (AIM) [77] and Feasibility of Intervention Measure (FIM) [77]. Although standardized thresholds for these measures have not been established, we will set our threshold for defining success as a mean of 4 or higher on the 5-point scale. Next, we will collect interviews with a subset of educators about their perceptions of the feasibility/acceptability of the de-implementation strategies as well as the feasibility/acceptability of cessation of detrimental feeding practices. This subset will include a purposive sample of educators based on their survey responses; we will randomly select from the top and bottom 25% teachers on the FIM scores to identify interview participants. We expect to interview 5 educators from each group. This will provide an in-depth and well-rounded perspective on feasibility in our study. Other measures collected in alignment with Re-AIM will serve as secondary outcomes. *Effectiveness* focuses on determining for whom the approach had positive impacts. We will assess child BMI and child dietary intake using Resonance Raman Spectroscopy (RRS) [78–83] to measure carotenoid intake and parent report of intake of WISE foods as well as nutrient-poor food consumption within the last month using a modified Food Frequency Questionnaire (FFQ) based on validated measures [84–86]. A passive consent process is approved for the ongoing trial to collect child BMI and RRS; we expect to follow the same protocol for this study and collect these data for up to 540 students. RRS will provide an objective measure of carotenoid intake [87], and analyses will use this as a within- and not between-person comparisons (i.e., avoiding comparisons across different skin pigmentations). This non-invasive method has been validated in preschoolers [79] and has several logistical advantages over invasive measures such as high-performance liquid chromatography in the community setting (e.g., higher participation, no trained phlebotomist required). Research suggests that, even among ethnically diverse samples, RRS can be an effective measurement tool when conducted on the palm of the hand [78]. For FFQ, all parents will be sent a form inviting them to participate. We will contact parents who respond for participation. Based on previous efforts with this approach, we expect to recruit 200 parents to complete FFQ assessments.

**Table 1:** Outcome measures to evaluate de-implementation approach

Construct	Instruments	Data collected
Effectiveness	BMI, RRS, FFQ	Impact on child weight and diet outcomes
De-adoption	Table Talk, BMER; AFC Strategies and Beliefs	Total number of detrimental and evidence-based feeding practices; scales include coercive control strategies, bribery with sweet foods, autonomy undermining, and social comparisons
De-implementation	Wise Fidelity; Qualitative interviews, AIM, FIM	Formative qualitative interviews to asses feasibility and acceptability of the de-implementation strategy, scales assessing acceptability and feasibility
Maintenance	AFC, Table Talk, BMER	Sustained impact on reported and observed behavior

*De-adoption* will be assessed with Table Talk [88] and the Food Intake module of the Building Mealtime Environments and Relationships (BMER) [89] inventory. These tools specifically measure the detrimental feeding practices targeted for de-implementation, as well as evidence-based ones targeted for adoption. Both are suited for live observations to record actual ECE behavior. These observations will occur three times across the intervention school year (fall, winter, spring) and once during the maintenance school year (fall). Each class (lead and assistant ECE) is observed twice at each observation period—once during a WISE lesson and once during the lunch mealtime for a total of 8 observations per class. Observers arrive in time to reduce reactivity before the observation time and then stay throughout the duration of the observation period (i.e., until the WISE lesson ends or the last child has the opportunity to eat).

Additionally, we will collect the About Feeding Children (AFC) Mealtime Strategies and Beliefs survey [90], a self-report measure of ECEs’ beliefs and intended practices. The survey includes scales to assess verbal (“I tell the children if they have not eaten enough”) and structural (e.g., classroom rule that children must take at least one bite) strategies that ECEs use when feeding children. In analyses, we will examine differences for ECEs who participated in both the EBQI sessions and the full-scale intervention.

Training protocols for the observational assessments (Table Talk and BMER) were refined in our previous work [88, 91]. First, data collectors complete a 2-h, in-person training that describes the logistics of completing observations, including discrete integration into the classroom visits to minimize reactivity. Second, data collectors learn about the intent of each observational item and code a video of a lunch as a group. Third, data collectors watch and code videos (at least 4 additional) on their own and review their answers with the PI or project RA, who have detailed answer keys. Data collectors must demonstrate 85% reliability against a video observation and live observation with the PI or project RA (i.e., established gold standard observers).

*Implementation* focuses on quality of delivery of and understanding why certain results were achieved (i.e., feasibility). To assess implementation, we will use the WISE fidelity measure [64]. This instrument is rated on a 1 to 4 scale, with 4 representing the highest level of fidelity. Each core component is assessed with 2 items, and overall scores on the fidelity form are created by summing scores across items. *De-implementation* strategy feasibility and acceptability will be assessed through surveys with the AIM and FIM with all participants as well as formative qualitative interviews with selected ECEs in Spring 2020 (*N* = 10) [92].

*Maintenance* is how well intervention components and their effects are maintained. Maintenance of the cessation of detrimental feeding and use of evidence-based feeding practices will be assessed in the subsequent school year. This is important as preventing reversal to routine habits is a challenge for de-implementation efforts [51]. We will be able to model maintenance variability.

### Analysis

#### Aim 1

We will use an online platform and database server to collect and store the EBQI panel’s ratings of importance and feasibility. In real time, we will query the database [93] to plot potential strategies by their rated importance (*x*-axis) and feasibility (*y*-axis) [76]. Strategies above the mean for both criteria will be considered for inclusion in the de-implementation approach.

After each EBQI session, research team members will write memos documenting what they observed and heard, what was resolved, and what remains undecided. The research team will discuss these and meeting minutes to guide the subsequent EBQI sessions and to inform development of the de-implementation training and support materials. The research team will also assimilate panel feedback, translate it to actionable plans, and develop the next iteration of materials that need input from the panel. Qualitative information from meeting minutes and audio recordings will be analyzed using directed content analysis [94] relative to the main goals of the EBQI process (e.g., matching barriers/facilitators to de-implementation strategies). After data from the final EQBI meeting are analyzed, the de-implementation approach will be ready for the proof-of-principle study in Aim 2.

#### Aim 2

To assess the feasibility and acceptability of our de-implementation approach, transcripts of qualitative interviews with ECEs will be coded [95]. Two coders will independently analyze the content and then resolve any differences together. These findings and feedback from the EBQI panel will be used to optimize the de-implementation approach in an iterative fashion to arrive at a fully-specified approach for a future large-scale trial.

Descriptive statistics on acceptability and feasibility (e.g., AIM, FIM) will provide valuable data to understand educators’ perceptions of the intervention. First, we will examine descriptive of the AIM and FIM measure to determine if the means reached our threshold for defining success (4 or higher out of 5). Next, we will compare item summary scores across participant characteristics to examine for potential patterns. We will focus on the following: (a) investigating variance in outcomes, (b) examining confidence intervals, and (c) assessing for the presence of practically-relevant effects [96–98]. Our targeted sample size will be adequate to provide useful information toward these objectives, pragmatic for recruitment, and consistent with sample sizes of other feasibility studies conducted in early care and education settings [97–101]. We will also use combined data from this study and the ongoing trial (i.e., Table Talk, AFC, BMI, RRS) to compare the 3 conditions ([1] basic WISE, [2] enhanced WISE, and [3] enhanced WISE with de-implementation support) on the targeted outcomes. We will compare confidence intervals, effect sizes, and variance in outcomes across the 3 conditions.

Comparison of data across the three conditions will inform our decision for progression of our de-implementation approach to a full trial. Criteria include (a) similar levels of acceptability and feasibility of the intervention across conditions, (b) qualitative feedback to support moving forward with the de-implementation approach, and (c) a trend toward improved change in feeding practices of the de-implementation condition compared to the implementation only conditions. That is, we would not progress to a full de-implementation trial if educator perceptions of acceptability and feasibility were markedly lower in the de-implementation study compared to the implementation only conditions or if qualitative feedback indicated notable concerns with the de-implementation strategies. Further, we desire to see greater pre to post reductions in use of detrimental feeding practices for the de-implementation condition compared to the implementation conditions to move forward to a full trial.

## Discussion

This study will produce a de-implementation approach that has a basis in the Niven model [56], is reflective of the culture, needs, and desires of ECEs while being consistent with views of administrators. To our knowledge, this will be the first study to apply the EBQI process to de-implementation rather than implementation and may be broadly applicable to the field of IS. Beyond our primary focus on establishing the feasibility and acceptability of our de-implementation approach, the study will provide preliminary data toward 3 predicted outcomes: (1) decreased detrimental feeding practices by ECEs, (2) increased adoption of and fidelity to WISE, and (3) improved child dietary outcomes. These results are expected to contribute to the uptake and sustainability of mealtime interventions to improve the diets of young children. That is, this study will focus on improving the environment in which food is consumed through decreasing detrimental food practices by the ECEs, regardless of what food is available to the children. This focus should facilitate program impact in centers that with a variety of food landscapes, including the two targeted in the current study—one which provides breakfast and lunch for the children and the other which allows for children to either bring their own food or eat in the cafeteria. Results will also apply broadly to the field of IS by informing processes for developing de-implementation approaches in a community, rather than clinical, setting. Thus, this study addresses a significant scientific question with an important public health impact.

Practical considerations for this project include planning for successful stakeholder engagement and addressing the lack of randomization in our study. To improve rapport with ECEs engaged in the EBQI process, we are including mentor panel members from a previous EBQI panel. We expect that including members of similar backgrounds and experiences (including EBQI experience) will increase comfort and trust among new members. These individuals will share their experiences and mentor new panel members. This is a unique innovation for EBQI, and we will share our evaluation of this process with the field. Although the limited number of sites in this study precludes randomization and blinding, our preliminary data (minimum of 2 observations per classroom) suggest that all classrooms have clear room for improvement in detrimental feeding and WISE fidelity. We will have baseline data for each classroom prior to the start of the de-implementation approach, allowing us to examine change from baseline. Finally, participant expectancy could threaten the validity of classroom observations. To counter this, we will use extended assessment periods (i.e., arriving 15 min early to allow adjustment) as well as multiple observation occasions and multiple methods of assessment (i.e., observed and self-reported feeding).

## Conclusions

The de-implementation approach developed in this study will address detrimental feeding practices and inform de-implementation efforts of other practices in other settings. Specifically, the lessons learned in this study will have potential for applicability to future de-implementation efforts of practices that are long-held and culturally ingrained in community settings. Further, the strategy developed in this study may be useful for school-age programs, after school care, and de-implementation aimed at parents.

## Data Availability

The datasets used for this current study will be available from the corresponding author on reasonable request.
